# The *Enterococcus faecalis* virulence factor ElrA interacts with the human Four-and-a-Half LIM Domains Protein 2

**DOI:** 10.1038/s41598-017-04875-3

**Published:** 2017-07-04

**Authors:** Alexandre Jamet, Rozenn Dervyn, Nicolas Lapaque, Francesca Bugli, Naima G. Perez-Cortez, Hervé M. Blottière, Jean-Claude Twizere, Maurizio Sanguinetti, Brunella Posteraro, Pascale Serror, Emmanuelle Maguin

**Affiliations:** 1 Micalis Institute, INRA, AgroParisTech, Université Paris-Saclay, 78350 Jouy-en-Josas, France; 2 Institute of Microbiology, University Cattolica del Sacro Cuore, Rome, Italy; 3 GIGA Proteins signalisation and interaction, University of Liege, Liege, Belgium; 4 Institute of Public Health, Section of Hygiene, Universita Cattolica del Sacro Cuore, Rome, Italy; 5 INRA, Unité d’Immuno-Allergie Alimentaire, iBiTecS/SPI, Gif-sur-Yvette, France

## Abstract

The commensal bacterium *Enterococcus faecalis* is a common cause of nosocomial infections worldwide. The increasing prevalence of multi-antibiotic resistant *E. faecalis* strains reinforces this public health concern. Despite numerous studies highlighting several pathology-related genetic traits, the molecular mechanisms of *E. faecalis* virulence remain poorly understood. In this work, we studied 23 bacterial proteins that could be considered as virulence factors or involved in the *Enterococcus* interaction with the host. We systematically tested their interactions with human proteins using the Human ORFeome library, a set of 12,212 human ORFs, in yeast. Among the thousands of tested interactions, one involving the *E. faecalis* virulence factor ElrA and the human protein FHL2 was evidenced by yeast two-hybrid and biochemically confirmed. Further molecular characterizations allowed defining an FHL2-interacting domain (FID) of ElrA. Deletion of the FID led to an attenuated *in vivo* phenotype of the mutated strain clearly indicating that this interaction is likely to contribute to the multifactorial virulence of this opportunistic pathogen. Altogether, our results show that FHL2 is the first host cellular protein directly targeted by an *E. faecalis* virulence factor and that this interaction is involved in Enterococcus pathogenicity.

## Introduction

*Enterococcus faecalis* belongs to the human core intestinal microbiota, *i.e* the limited number of species that are shared by more than 50% of the population^1^. Although it is a common inhabitant of our intestinal tract, *E. faecalis* is also a major opportunistic pathogen for critically ill or immuno-compromised patients^2, 3^. Indeed, *E. faecalis* is ranked among the most common nosocomial pathogens worldwide infecting the bloodstream and urinary tract. Understanding the molecular mechanisms of *E. faecalis* pathogenicity is therefore crucial to design new therapeutic approaches to treat enterococcal infections even when strains resistant to antibiotics are involved. *E. faecalis* does not harbour virulence factors such as effectors and toxins identified in Gram-positive pathogens. Instead, the pathogenicity of *E. faecalis* relies on a multifactorial process that involves biofilm formation, antibiotic^4, 5^ and stress resistances^6–8^, adhesion to host cells and intracellular survival^3^.

Several *E. faecalis* surface exposed proteins involved in different processes are likely to contribute to bacterial pathogenicity *e.g*., adhesion to host cells, host cell invasion and bacterial protection against host defenses. Except for the enterococcal fibronectin-binding protein A (EfbA)^9^, most of these surface exposed proteins possess a surface-exposure motif (anchoring or retention motif) such as the WxL domain like of the enterococcal leucine-rich protein A (ElrA)^10^ or a LPxTG-anchored domain as for the aggregation substance (AS) proteins^11^, adhesin to collagen (Ace)^12^, Ebp pilus^13, 14^, the FSS^15^, and PrgB^16^ proteins.

These *E. faecalis* surface exposed proteins were reported as targeting different host extracellular matrix proteins. Among the members of the AS (aggregation substances) proteins, Asa1 interacts with intestinal epithelial cell lines, macrophages and neutrophils via its binding to CD11b/CD18 integrin, fibronectin and thrombospondin^17, 18^. Ace is characterized by adjacent immunoglobulin-like folds that define the family of Microbial Surface Components Recognizing Adhesive Matrix Molecules (MSCRAMMs) and it interacts with collagen type I and laminin^12, 19^. The anchorless EfbA protein binds fibronectin^9^. Finally, Ebp pili adhere to fibrinogen, collagen, and to platelets^14, 20^. Overall, these proteins or complexes seem to have multifunctional roles, suggesting that they may interact with different host-cell unknown components through different functional domains.

To extend our knowledge on the mechanisms developed by *E. faecalis* to interact with its host, we used a systematic high-throughput yeast two-hybrid (HT-Y2H) approach to identify protein interactions between selected surface protein of this bacterium and the human proteome. Such screening approaches were successfully used with pathogenic microbes, including bacteria such as *Brucella* ^21^ or *Pseudomonas syringae* ^22^ and viruses such as Epstein-Barr^23^, Hepatitis C^24^, HTLVs^25^, or dengue fever virus^26^. Here, we report the systematic screening for interactions between 23 selected *E. faecalis* proteins and the human ORFeome v3.1 (hORFeome), and the identification of a specific interaction between the *E. faecalis* ElrA and the human FHL2, a member of the Four-and-a-Half LIM Domains Protein family. Disruption of this interaction led to an impairment of the *in vivo* pathogenic phenotype strongly suggesting a role of this newly identified interaction in the *E. faecalis*-host interplay.

## Results

### Selection of 23 *E. faecalis* ORFs

**Figure 1:**
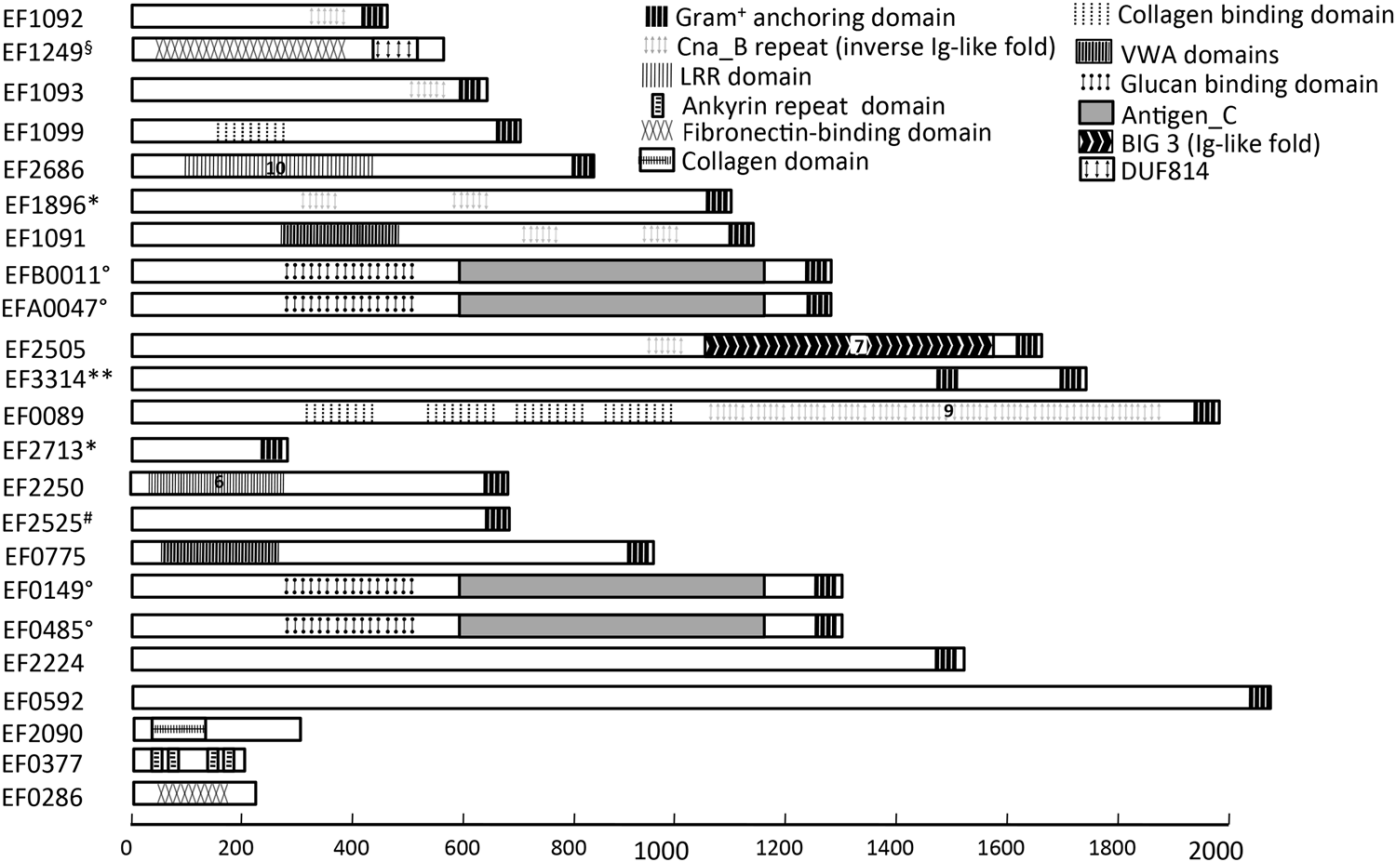
Representation of the *E. faecalis* set of proteins with their domains. The proteins are designed by their EF numbers; some of them were also published with the following names: EF1092: EbpB, EF1093: EbpC, EF1099: Ace, EF2686: ElrA, EF0775: EbsG, EF1896: Fss3, EF1091: EbpA, EFB0011: PrgB, EFA0047: Asa1, EF0149: PrgB-like, EF0485: AS, EF0089: Fss1, EF1249: EfbA. Additional information on expression or homology of the protein are shown as follows: *enriched in clinical isolates; **induced in the gastrointestinal tract; °aggregation substance-like; ^#^phage-like protein; ^§^FbpA like protein.

We defined three criteria based on previous publications in the field of host-bacteria interactions to select 23 proteins expressed by *E. faecalis* and that could promote interaction with the host namely proteins harbouring a putative surface-exposure motif (anchoring or retention motif) and/or a eukaryotic-like domain and proteins already described as involved in *E. faecalis* virulence (Table 1 and Fig. 1).

**Table 1:** Description of the 23 *E. faecalis* selected ORFs.

EF gene	Other name	Vir factor	Domains	Retention
EF1092	EbpB	69	Cna_B repeat domain	LPxTG
EF1249	EfbA (FbpA-like)	9, 70	Fibronectin binding d (pfam05833)	—
EF1093	EbpC	9	Cna_B repeat domain	LPxTG
EF1099	Ace	71	Collagen binding domain	LPxTG
EF2686	ElrA	10	LRR (COG4886)	WxL
EF1896	Fss3	15	Cna_B repeat domain	LPxTG
EF1091	EbpA	69	Willebrand factor (vWA) Cna_B repeat domain	LPxTG
EFB0011	PrgB	16	Glucan binding dom (pfam08363) Antigen_C (pfam16364)	LPxTG
EFA0047	Asa1	17	Glucan binding dom (pfam08363) Antigen_C (pfam16364)	LPxTG
EF2505	Fss2	15	BIG3 domain Cna_B repeat domain	LPxTG
EF3314	—	72		LPxTG
EF0089	Fss1	15	Collagen binding dom (pfam5737) Cna_B repeat domain	LPxTG
EF2713	—	no	*E. faecalis* specific protein	LPxTG
EF2250	—	no	LRR (COG4886)	WxL
EF2525	(phage like prot)	no	*E. faecalis* specific protein	LPxTG
EF0775	EbsG	no	Willebrand factor (vWA)	LPxTG
EF0149	PrgB-like	no	Glucan binding dom (pfam08363)Antigen_C (pfam16364)	LPxTG
EF0485	AS (agreg subs)	no	Glucan binding dom (pfam08363) Antigen_C (pfam16364)	LPxTG
EF2224	—	no		LPxTG
EF0592	—	no		LPxTG
EF2090	—	no	Collagen domain (pfam01391)	—
EF0377	—	no	Ankyrin domains (pfam00023)	—
EF0286	—	no	Fibronectin binding (pfam07299)	—

Based on sequence analysis, we first selected 12 surface exposed proteins known to be involved in *E. faecalis* virulence: 10 containing an LPxTG anchoring domain (EbpA, EbpB, EbpC, Ace, Fss1, Fss2, Fss3, EF3314, Asa1 and PrgB), 1 containing a WxL retention domain (ElrA) and 1 anchorless protein (EfbA). It is well established that cell surface anchored proteins are involved in bacterial-host interactions and that in pathogens, they are often required for the first steps of infection by promoting the interaction with the host targeted cells^27–29^. Moreover, these surface exposed virulence factors have either prokaryotic or eukaryotic-like domains, which can be involved in interaction with the host components (Table 2). Based on these data, we selected 11 additional *E. faecalis* proteins that have surface anchors and/or eukaryotic-like domains but which are not integral membrane proteins that were excluded to avoid false positive hit due to hydrophobic regions in the Y2H screen^30, 31^. The additional selected *E. faecalis* proteins were: EF2525, EF2713, EF2250, EbsG, Prg-B-like, EF0485, EF2224, EF0592, EF2090, EF0377, EF0286.

**Table 2:** Strains and plasmids used in the current study.

Strain	Relevant Genotype or Description	Reference or Source
*E. coli*		
TOP10	F- mcrA Δ(mrr-hsdRMS-mcrBC) φ80lacZΔM15 ΔlacX74 nupG recA1 araD139 Δ(ara-leu)7697 galE15 galK16 rpsL(StrR) endA1 λ-	Invitrogen corporation
DB3.1	*ccdB* resistant cloning strain F- gyrA462 endA1 glnV44 Δ(sr1-recA) mcrB mrr hsdS20(rB-, mB-) ara14 galK2 lacY1 proA2 rpsL20(Smr) xyl5 Δleu mtl1	Invitrogen corporation
ER2566	F- λ- fhuA2 (lon) ompT lacZ::T7 gene 1 gal sulA11 Δ(mcrC-mrr)114::IS10 R(mcr-73::miniTn10-TetS)2 R(zgb-210::Tn10)(TetS) endA1 (dcm)	Invitrogen corporation
*S. cerevisiae*		
Y8800	*MAT*a leu2–3,112 trp1–901 his3Δ200 ade2–101 gal4Δ gal80Δ cyh2R GAL2::ADE2 GAL1::HIS3@LYS2 GAL7::LacZ@met2	62
Y8930	*MATα* leu2–3,112 trp1–901 his3Δ200 ade2–101 gal4Δ gal80Δ cyh2R GAL2::ADE2 GAL1::HIS3@LYS2 GAL7::LacZ@met2	62
*E. faecalis*		
V583 (VE14002)	Hospital strain, Van^R^	73
OG1RF (VE14001)	Plasmid-cured derivative from human oral cavity, Fus^r^ Rif^r^;	74
VE14201	OG1RF strain, Δ*elrA* mutant	10
VE18355–3	OG1RF strain, *elrA*ΔFID mutant 1	this study
VE18355-4	OG1RF strain, *elrA*ΔFID mutant 2	this study
Plasmid	**Description**	**Reference or source**
pDONR223	Spectinomycin resistant cloning vector	Invitrogen corporation
pDestAD-GW-CYH	Single copy plasmid allowing Gal4 activation domain (AD) hybrid proteins fusion	60
pDestDB-GW	single copy plasmid allowing Gal4 DNA binding domain (DB) hybrid proteins fusion	60
pGEX-2TK-GW	GST fusion vector Gateway-compatible	21
pCMVHA-GW	pCMVHA (Clontech) Gateway-compatible	21
pCMVmyc-GW	pCMVmyc (Clontech) Gateway-compatible	64
pV1899	pCMV 5′ Triple Flag Nterm Gateway-compatible	61
pGEM-T Easy	T vector with f1 *ori*R, Ap^R^	Promega corporation
pG^+^host9		^66^
pDONR-EF2686	pDONR223 carrying EF2686 (*elrA*)	this study
pDestDB-EF2686	pDestDB carrying EF2686 (*elrA*)	this study
pDONR-FHL1	pDONR223 carrying FHL1 (hORF 11050@D04)	35
pDONR-FHL2	pDONR223 carrying FHL2 (hORF 31042@A08)	36
pDONR-FHL3	pDONR223 carrying FHL3 (hORF 11031@C01)	35
pDONR-FHL3-Cter	pDONR223 carrying FHL3 (hORF 11018@E03)	35
pDONR-FHL5	pDONR223 carrying FHL5 (hORF 11050@F02)	35
pDONR-GOPC	pDONR223 carrying GOPC (hORF 11063@H10)	35
pDONR-CDC23	pDONR223 carrying CDC23 (hORF 11088@H06)	35
pDONR-EFXXXX	pDONR223 carrying EFXXXX	this study
pDONR-ElrA_abc-xyz_	pDONR223 carrying the *elrA*fragment (corresponding to amino acid abc to xyz) amplified with primers ElrA_FX and ElrA_RX	this study
pDB-ElrA_abc-xyz_	pDestDB carrying *elrA* transfered by LR reaction from pDON-ElrA_abc-xyz_	this study
pGEX-ElrA_27-723_	pGEX-2TK-GW carrying the *elrA* fragment transfered from pDONR-ElrA_27–723_	this study
pGEX-ElrA_27–473_	pGEX-2TK-GW carrying the *elrA* fragment transfered from pDONR-ElrA_27–473_	this study
pGEX-ElrA_474–606_	pGEX-2TK-GW carrying the *elrA* fragment transfered from pDONR-ElrA_474–606_	this study
pCMVmyc-ElrA_27–723_	pCMVmyc-GW carrying the *elrA* fragment transfered from pDONR-ElrA_27–723_	this study
pCMVmyc-ElrA_27–473_	pCMVmyc-GW carrying the *elrA* fragment transfered from pDONR-ElrA_27–473_	this study
p3flag-FHL2	pV1899-GW carrying FHL2 transfered from pDONR-FHL2	this study
pCMVHA-FHL2	pCMVHA-GW carrying FHL2 transfered from pDONR-FHL2	this study
pGEM-T-*elrA*ΔFID	pGEM-T Easy, with fragment *elrA*ΔFID	this study
pG^+^host9-*elrA*ΔFID	pG^+^host9, with *Eco*RI fragment of pGEM-T-*elrA*ΔFID	this study

Altogether, we selected a set of 19 potentially surface exposed proteins (Fig. 1 and Table 1): 16 proteins contain an LPxTG anchoring domain, 2 proteins have a WxL retention domain and 1 is an anchorless protein. LPxTG-proteins EF2525 and EF2713 exhibiting neither predicted domain nor homology with other bacterial proteins were selected and can be considered as *E. faecalis* specific proteins. The 17 other proteins contained either prokaryotic or eukaryotic-like domains likely to promote protein-protein interactions. The prokaryotic domains are: (i) the Glucan binding domain and Antigen_C (PrgB, Asa1, PrgB-like and EF0485), (ii) a collagen- or fibronectin-binding domain (Fss1, Ace and EfbA) and (iii) the Leucine rich repeat (LRR) domain (ElrA and EF2250) interaction. Regarding the eukaryotic-associated motif, we found the von Willebrand factor type A (vWA) domain that was originally found in the blood coagulation protein von Willebrand factor (vWF), a signature of adhesion in eukaryotes present in EbsG and EbpA, two Ig-like fold domains (i) the bacterial immunoglobulin-like BIG3 domain detected in EF2505 and (ii) the Cna_B repeat detected in 6 *E. faecalis* proteins (EbpA, EbpB, EbpC, Fss1, Fss2 and Fss3). Note that the Fss proteins 1 to 3 have been shown to bind fibrinogen^15^. Three proteins exhibiting the ankyrin repeat, fibronectin binding or collagen eukaryotic-like domains were also selected. EF0377 displays 4 ankyrin domains. The ankyrin domains have been shown to promote specific interactions with host proteins leading to the impairment of the targeted molecule activity. Many examples demonstrated the role of ankyrin repeat bacteria effectors in the hijacking of eukaryotic functions by the pathogen during the infection process^32^. EF0286 and EF1249 have a fibronectin-binding domain (pfam07299 that enables adhesion of the bacteria to the host tissues. EF2090 exhibits collagen domains involved in protein interactions of fiber structures.

The selected set of 23 *E. faecalis* proteins, harbour particular domains that are likely to be involved in the interaction of the bacterium with the host and are summarized in Table 1 and Fig. 1.

### Identification of ElrA FHL interactions using HT-Y2H

The interactions between the 23 selected *E. faecalis* putative virulence factors and the Human proteome were tested using the yeast two-hybrid approach^33^. The 23 *E. faecalis* ORFs were amplified by nested PCR amplification^34^, cloned in the pDONR223 using the Gateway system and transferred in the pDestDB-GW vector (see Material and Methods) prior to interactomic assays. Each selected *E. faecalis* ORFs was tested with the human ORFeome v3.1 containing 12,212 human ORFs^35, 36^. Among the 280,876 tested interactions, a single protein out of the 23 tested gave positive hits. The candidates interacting pairs of proteins were identified, revealing ElrA^10^, interacted with 3 human ORFs: FHL2 (31042@A08), GOPC (11063@H10) and CDC23 (11088@H06). The interactions were individually validated by mating the yeast strains carrying ElrA fused to the Gal4 DNA binding domain (BD) and the human ORFs flanked by the Gal4 Activating Domain (AD) and growing them on medium selecting for strong interactions. The interaction of ElrA with FHL2 was the only one confirmed. FHL2 is a member of the four-and-a-half-LIM protein family, known to participate in many cellular processes^37^ and including FHL1, FHL2, FHL3, FHL4 and FHL5/ACT in humans. The LIM domain is a highly conserved double zinc-finger motif^38^.

**Figure 2:**
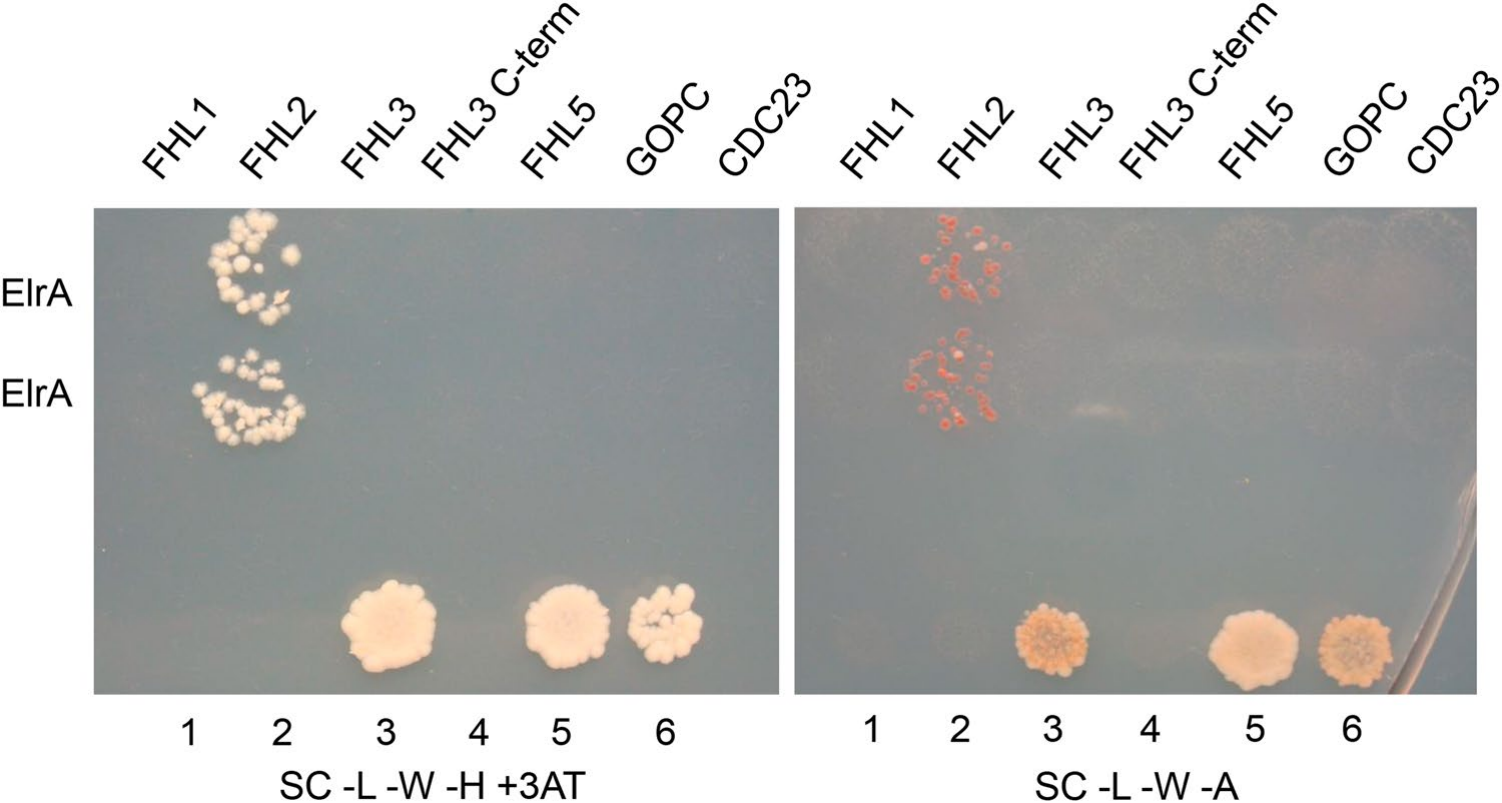
ElrA and FHL family interaction assays. Y2H interaction matrix between ElrA and FHL1 (11050@D04), FHL2 (31042@A08), FHL3 (11031@C01), a truncated variant of FHL3 containing 1/2 LIM2, LIM3 and LIM4 domains (11018@E03), FHL5 (11050@F02), GOPC (11063@H10) and CDC23 (11088@H06). Mating yeasts were replicated on high stringency selective media *i.e*., minimal medium lacking leucine, tryptophan, histidine and containing 5 mM of 3-aminotriazole (SC-L-W-H + 3AT) or minimal medium lacking leucine, tryptophan and adenine (SC-L-W-A). The yeast strains used as controls are carrying the following vectors 1, pPC97-CYH (DB-empty) and pPC86 (AD-empty), 2, DB-RB1 and AD-E2F-1, 3, DB-fos and AD-jun, 4, pPC97 (DB-empty) and pPC86 (AD-empty), 5, DB-DP1 and AD-E2F1, 6, DB-FRM-3 and AD-PKc_like.

The hORFeome contains 5 different FHL sequences: FHL1 843 ncl (11050@D04), FHL2 840 ncl (31042@A08), FHL3 843 ncl (11031@C01), FHL3 519 ncl (11018@E03) a truncated variant of FHL3 and FHL5 855 ncl (11050@F02). In the same experimental set-up we tested the individual interaction of each clone with the ElrA protein. In stringent selective conditions, the interaction was only detected for FHL2 (Fig. 2). As no interaction was detected between ElrA and the other FHL family members, we concluded that in the Y2H assay, ElrA interacts specifically with FHL2.

### Identification of the minimal ElrA domain interacting with FHL2

**Figure 3:**
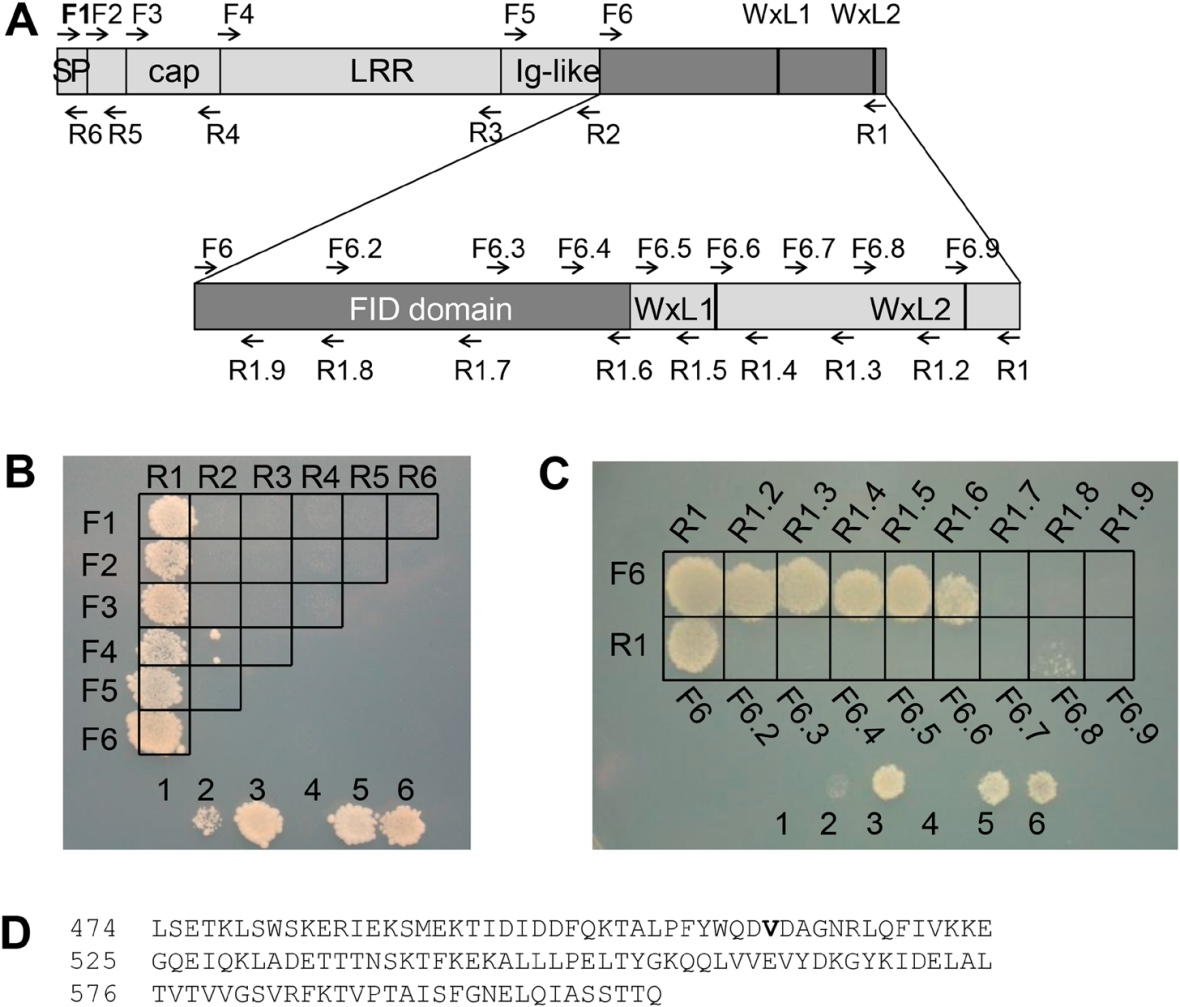
Determination of the ElrA minimal domain interacting with FHL2. (**A**) Schematic representation of ElrA with a zoom on the C-terminal region, known as the WxL domain. The forward and reverse primers used to generate the ElrA fragments are shown as F and R, respectively. The correspondence between the position of primers and the ElrA amino acids is detailed in Table S1. Symbols: SP, signal peptide, cap, cap domain, LRR, Leucine rich repeat domain, Ig-like, Immunoglobulin-like domain, and WxL, anchor domain with the WxL1 and WxL2 motifs are represented as thick dark bars. (**B**) Y2H interaction matrix between FHL2 and the 21 ElrA fragments generated with the primers F1 to F6 (lanes) and R1 to R6 (columns). Accordingly, the first box on the top left of the figure corresponds to the F1-R1 amplicon *i.e*. the full-length ElrA. (**C**) Y2H interaction matrix between FHL2 and the 17 ElrA fragments generated with the F6 to F6.9 and the R1 to R1.9 primers. The minimal FHL2 Interacting Domain (FID) spans from primers F6 to R1.6. (**D**) Amino acid sequence of the FID domain. In (**B**) and (**C**), the yeast strains used as controls are containing the following vectors 1, pPC97-CYH (DB-empty) and pPC86 (AD-empty), 2, DB-RB1 and AD-E2F-1, 3, DB-fos and AD-jun, 4, pPC97 (DB-empty) and pPC86 (AD-empty), 5, DB-DP1 and AD-E2F1, 6, DB-FRM-3 and AD-PKc_like.

To identify the domain of ElrA involved in the interaction with FHL2, several deletions of the ElrA protein (Fig. 3A) were generated using primers located at the limits of the 5 domains previously described^10^: the signal peptide (aa 1 to 26), the cap domain (aa 61 to 141), the Leucine Rich Repeat domain (aa 142 to 387), the Immunoglobulin-like domain (aa 388 to 473) and the WxL domain (aa 474 to 723). The 21 ElrA-fragments resulting from the amplifications were fused to the Gal4-DB in the pDestDB-GW vector (Table S1) and tested for their ability to interact with FHL2 fused to the Gal4-AD in yeasts. Interestingly, all the fragments containing the WxL domain interacted with FHL2 whereas no interaction was observed with the other 15 ElrA truncated mutants (Fig. 3B). The WxL domain contains 2 WxL motifs at position 628–630 (WxL1) and 712–714 (WxL2) and is responsible for the interaction with the peptidoglycan and the bacterial cell surface retention of ElrA^10^.

We further investigated whether the two WxL motifs were responsible for the interaction with the FHL2 human protein. Sixteen sub-fragments of the WxL domain (aa 474 to 723) were amplified (Fig. 3A) and cloned in fusion with Gal4-DB. We tested the interaction with FHL2 fused to the Gal4-AD; the region spanning from the residues 474 to 606 interacted with FHL2 defining the minimal FHL2 interaction domain (FID) of ElrA (Fig. 3C and D). Interestingly the 2 WxL motifs located between the aa 628 to 630 and the aa 712 to 714 are not involved in the interaction with FHL2. Consequently, the ElrA region that was previously called the WxL domain (aa 474 to 723) indeed corresponds to two different functional domains: (i) FID (residues 474 to 606) and the WxL domain (residues 607 to 723).

**Figure 4:**
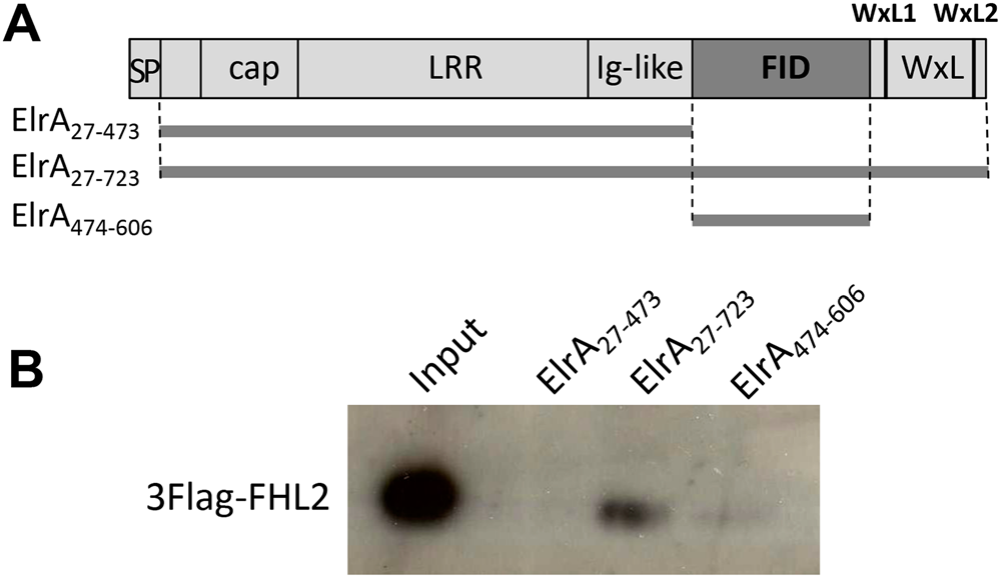
GST-pull down assay with ElrA and FHL2. (**A**) Schematic representation of the ElrA protein and of the 3 constructs used for the pull down. 1, ElrA_27–473_, 2, ElrA_27–723_, 3, ElrA_474–606_. Symbols: SP, signal peptide, cap, cap domain, LRR, Leucine rich repeat domain, Ig-like, Immunoglobulin-like domain, FID, FHL2 interacting domain, WxL, WxL anchor domain with the WxL1 and WxL2 motifs. (**B**) Pull-down assays, Western blot. The M2 anti-Flag antibody was used to detect the 3Flag-FHL2 from the HEK293T cells lysate pulled down by the GST-ElrA_27–473_ (lane 1), GST-ElrA_27–723_ (lane 2) or GST-ElrA_474–606_ (lane 3). 3Flag-FHL2 lysate (input) was used as a positive control to check the 3Flag detection. Full-length blot is presented in Figure S2.

GST pull-down and immunofluorescence microscopy were used as independent methods to confirm the FID/FHL2 interaction showed by Y2H. Three GST-ElrA fusions corresponding to (i) ElrA_27–723_ i.e., the full-length protein without its signal-peptide, (ii) ElrA_27–473_ i.e., the N-terminal part of ElrA without the FID and the WxL domains and (iii) ElrA_474–606_ corresponding to the FID were tested with a 3Flag tagged FHL2 in a pull-down assay (Fig. 4A). As expected, the interaction between FHL2 and ElrA_27–723_ was observed. Interaction of FHL2 also occurred to a minor extend with ElrA_474–606_ that contained only the FID, were observed (Fig. 4B).

**Figure 5:**
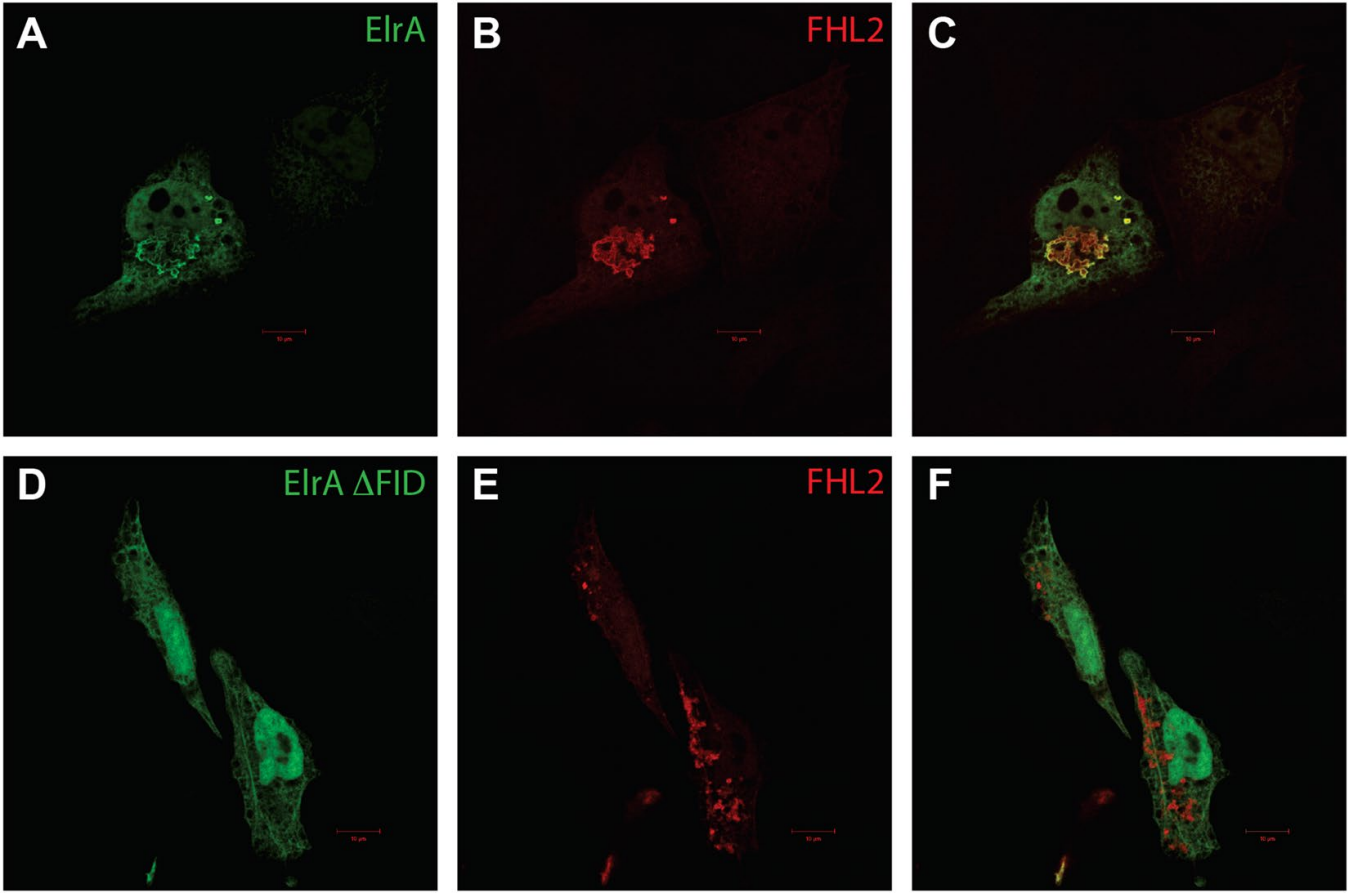
Cellular localisation of untruncated and FID-truncated forms of ElrA and FHL2. HA-FHL2 and myc-ElrA were produced after transfection of HeLa cells with the pCMVHA-FHL2 and pCMVmyc-ElrA_27–723_ or pCMVmyc-ElrA_27–473_ plasmids. A representative cell is shown for each form of myc-ElrA. In the left panels (A and D), is represented the localisation of the untruncated (**A**) and the FID-truncated ElrA (**D**) the FHL2 staining (anti-HA) is represented in the middle panels (**B** and **E**), and the right panels were generated by merging the anti-myc and anti-HA signals (C and F). Scale bars = 10 µm. Localization of HA-tagged FHL2 (**B**) is similar (**C**) to the localization of an untruncated myc-ElrA (A) whereas localization of HA-tagged FHL2 (**E**) is different (**F**) to the localization of a truncated myc-ElrA (**D**).

HeLa cells were transfected with plasmids encoding a HA-FHL2 and a myc-ElrA constructs coding for ElrA_27–723_ (containing FID, FID + ) or ElrA_27–473_ fragments (deleted for the FID, FID-). By confocal microscopy, a clear co-localisation was observed between FHL2 and the wild-type ElrA_27–723_ fusion protein, but not with the FID-truncated ElrA_27–473_ fragment. These data are consistent with an interaction of FHL2 with ElrA through the C-terminal part of ElrA in a cellular context (Fig. 5). Altogether these results are in agreement with a role of FID (aa 474 to aa 606) in the ElrA/FHL2 interaction.

### *In vivo* importance of the ElrA FHL2 Interacting Domain

**Figure 6:**
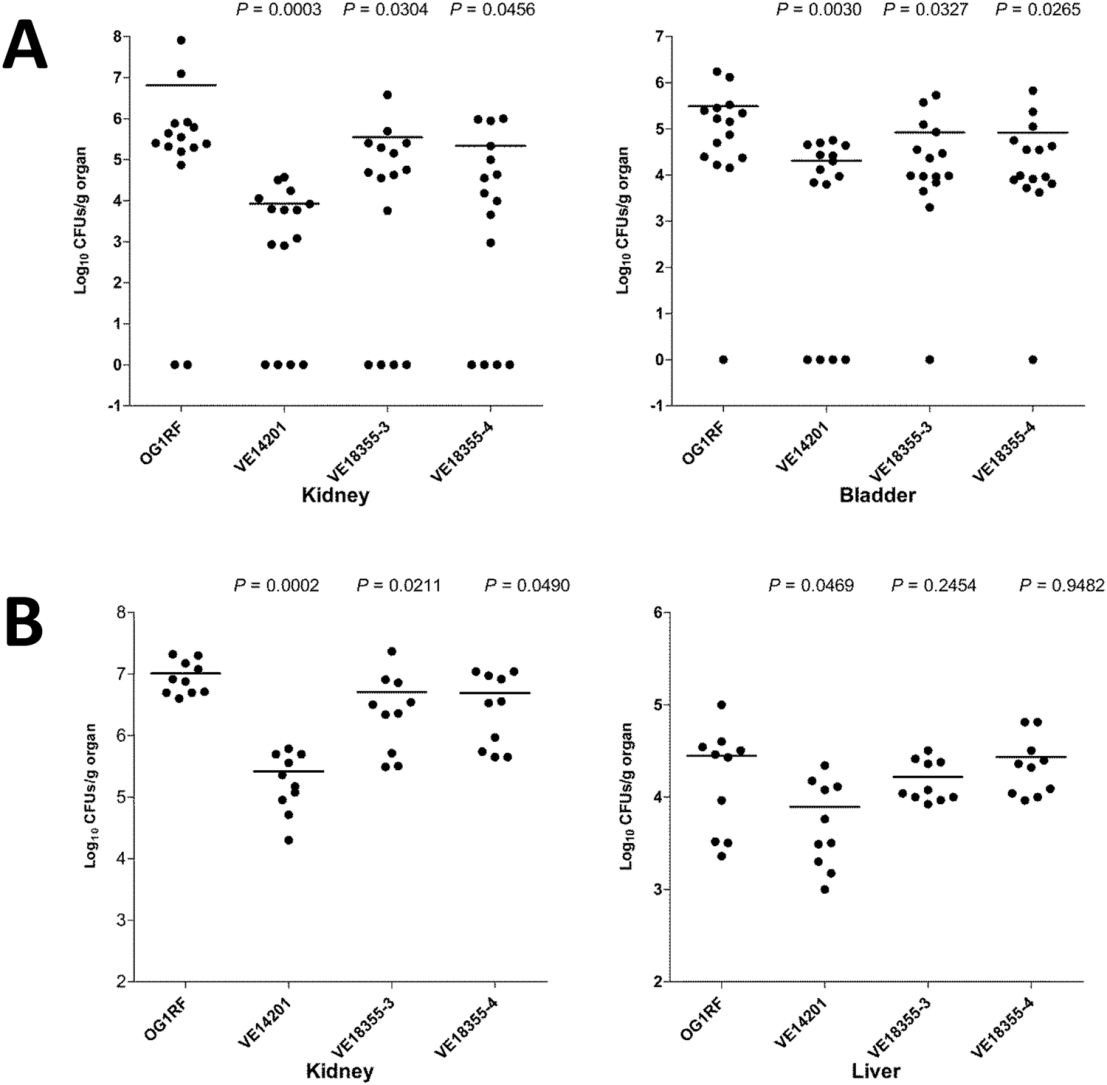
*In vivo* effect of the FID deletion in ElrA in two mice infection models. (**A**) Groups of 15 BALB/c mice were challenged transurethrally with the OG1RF wild-type, VE14201 Δ*elrA*, VE18355–3 and VE18355–4 *elrA*ΔFID strains. Data are expressed as the log_10_ colony-forming units (CFUs) recovered per gram of kidneys or urinary bladder homogenates collected 48 hours after the challenge. The log_10_ CFUs from both kidneys were combined and averaged. A value of 0 was assigned to the uninfected organs. Horizontal bars represent the geometric means. *P* values of less than 0.05 were considered to be significant. (**B**) Groups of 10 BALB/c mice were contrast to the ElrA-deficient strain infected with the OG1RF wild-type, VE14201 Δ*elrA*, VE18355–3 and VE18355–4 *elrA*ΔFID strains. Data are expressed as the log_10_ colony-forming units (CFUs) recovered per gram of kidneys or liver homogenates collected 7 days after the challenge. Horizontal bars represent the geometric means. *P* values of less than 0.05 were considered to be significant.

Identification of an ElrA domain that interacts with FHL2, led us to hypothesize that deletion of the FID domain may affect virulence as it would impair ElrA interaction with FHL2. To test this hypothesis and considering a potential tissue-specific interaction between ElrA and FHL2, we compared the virulence of a mutant strain deleted for the FID domain to the parental strain (OG1RF) and the Δ*elrA* mutant strain in two different mouse models: urinary tract (UTI) and systemic infection (SI) models. We constructed and tested two independent isogenic *elrA* mutants deleted in-frame of residues 474 to 606 that encompass the FHL2 interacting domain (*elrA*ΔFID). In the UTI model (Fig. 6A), the Δ*elrA* strain exhibited a decrease of 3 log units (P = 0.0003) in kidney and of 1 log unit decrease (P = 0.00304) in bladder compared to the WT strain, revealing that *elrA* contributes to pathogenesis of *E. faecalis* urinary tract infection. Interestingly, the organ bacterial loads upon infection with *elrA*Δ*FID* were intermediate between the values observed with the WT and those obtained with the Δ*elrA*-mutated strain, indicating that ElrA-FHL2 interaction is implicated in the ElrA-dependent pathogenesis in the urinary track. We then examined the virulence of the three strains in a systemic infection model post-infection intravenously (Fig. 6B). Strain Δ*elrA* showed significantly decreased burden of the liver (~ 1 log units) and kidneys (1.5 log unit) compared to the WT strain. In contrast, strain *elrA*Δ*FID* showed an intermediate phenotype and appeared to colonize the liver at a comparable level as the WT while it colonized kidneys at a significant lower level (~0.4 log units of reduction for each strain; *P* = 0.02 and *P* = 0.049 for the two tested strains) than the WT, supporting that *elrA* contributes to *E. faecalis* systemic infection and partially relies on the interaction of ElrA with FHL2. Altogether, these results indicated that the interaction between ElrA and FHL2 have a role in systemic and urinary infections.

## Discussion

Several bacterial pathogen effectors bind host proteins to hijack the host functions for their own benefits^39^. In Gram-positive bacteria, several bacterial virulence factors are located at the pathogen surface such as the internalins of *L. monocytogenes* ^27^. In this study, we selected and cloned 23 putative virulence factors of the *E. faecalis* opportunistic pathogen and tested their ability to interact with host proteins using the hORFeome v3.1^35, 36^. We identified a strong interaction between the virulence factor ElrA of *E. faecalis* and the human protein FHL2, and determined the minimal interacting domain of ElrA, named FID (FHL2-Interacting Domain). In addition, we showed that FID is partially involved in the ElrA-dependent virulence *in vivo*. To our knowledge, besides interactions with extracellular matrix proteins, FHL2 is the first intracellular host protein targeted by a virulence factor of *E. faecalis*.

In our Y2H experimental set-up using stringent conditions^33^, only strong interactions are detectable, this explains the low range of positive hits. In the selected surface proteins, ElrA is one of the proteins that is likely to have a transient interaction with the bacterial surface as its WxL domain promotes a non covalent interaction with the peptidoglycan^10^. Then ElrA could be considered both as a surface and as a secreted protein. For the proteins covalently bound to the bacterial surface (e.g. those with an LPxTG motif), the Y2H have shown limits in the discovery of surface-surface protein interactions. This could be due to the low level of posttranslational modifications in yeast while they are important for many cell surface proteins (e.g., O- or N-glycosylation for eukaryotic cell receptors^40^) or the absence of multimerisation in Y2H experiments while it is required for the activity of many eukaryotic surface receptors. For example, the detection of an interaction between the bacterial flagellin and the TLR5 homodimer receptor is not detectable using Y2H (A. Jamet personal data). However, the set of entry vectors encoding *E. faecalis* ORFs generated in this study, provides an interesting tool of easily transferable inserts to Gateway compatible vectors for other applications, such as the expression of an *E. faecalis* protein fused with tags that can be used for purification or tandem affinity purification allowing the purification of stable complexes to identify the partners involved^41^ or with reporters such as the GFP, which can be used to localize the protein^42^.

ElrA is an internalin-like protein that contains a signal-peptide, a leucine-rich repeat domain followed by an Ig-like domain and at the C-terminal end, a WxL domain, which interacts with the bacteria peptidoglycan^10^. It was demonstrated that: i) the inactivation of *elrA* significantly reduced *E. faecalis* virulence in a peritonitis model and that ElrA contributed to the bacterial intracellular persistence in macrophages and increased the host inflammatory response by stimulating *in vivo* the production of interleukin-6^10^. Overexpression of ElrA protects from phagocytosis *in vitro* and increases *E. faecalis* virulence a mouse peritonitis model^43^. It was also shown that *elrA* is the first gene of an operon poorly transcribed under laboratory growth conditions but induced in mice during infection^10^. However, so far no ElrA ligand was identified. In this study, we identified a physical interaction between ElrA and FHL2, a member of the four-and-a-half-LIM-only family proteins. This interaction happens *in vitro* and impacts the *in vivo* infectious process.

The members of the four-and-a-half-LIM-only protein family are involved in many physiological processes probably through their ability to interact specifically with various proteins. Due to their interacting ability, the LIM-only proteins and by extension all proteins containing LIM domain(s), are considered as adaptors or scaffolding proteins^44^. To date in the host-microbe interactions context, a specific interaction has been shown between Tax1 a protein of the human T-cell leukemia virus 1 (HTLV-1) and FHL3^45^. Our study is the first report describing an interaction between a bacterial protein and a specific member of the FHL family. FHL2 has a strong homology with the other members of the four-and-a-half-LIM-only protein family, which includes FHL1, FHL3, FHL4 and FHL5. The human FHL2 amino acid sequence is 48.2% identical with FHL1, 53.4% with FHL3, 48.4% with FHL4, and 59.1% with FHL5. Although all FHLs could promote protein-protein interactions^37^, we demonstrated that ElrA interacts specifically with FHL2. FHL2 is involved in heart physiology, bone formation, muscular function and placental development. In addition, FHL2 is involved in a wide range of various cellular processes such as cytoarchitecture, cell adhesion, cell survival, cell mobility, signal transduction and other processes through the regulation of gene expression^46^. FHL2 enhances the transcription of the tumor necrosis factor (TNF) receptor-associated factor 6 (TRAF6) through the stabilization of NF-κB after activation^47^. FHL2 is enzymatically inactive but has been shown to interact with more than 100 proteins through its LIM domains^48^ although, the comparison of various FHL2 partners do not allow the identification of a consensus interacting sequence. The FHL2 partners are components of crucial signaling pathways (protein kinases, transcription factors, receptors or receptors interacting proteins), proteins known to modulate RNA splicing, DNA replication and repair. The broad range of cellular partners and involvement in cellular processes make FHL2 an interesting target for a pathogenic bacterium to ensure its survival. However, the clear picture of the cellular mechanisms underlying and rising from the FHL2/ElrA interaction remains to be found.

*Enterococcus* are traditionally considered as commensal extracellular bacteria, during its infectious process it can be found intracellular within urothelial cells shed from the bladder of patients^49^ or surviving for long periods into macrophages^50–53^. Moreover, several laboratories showed that *E. faecalis* escapes the phagocytic vacuole to avoid lysosomal elimination^51–53^. At late stages of infections, intracellular *E. faecalis* activates phosphatidylinositol 3-kinase signaling to block apoptotic cell death in macrophages^52, 53^. As FHL2 is detected in the cytoplasm, we could anticipate that FHL2/ElrA interaction occurs during bacterial intracellular survival. However, as ElrA is non-covalently attached to the bacterial peptidoglycan^10^, we cannot rule out the possible release of ElrA directly into the cytoplasm of the host cell during the early interaction steps. What are the incidences or signalling pathways targeted by the ElrA/FHL2 interaction remain unknown. The interaction with ElrA could even compete with an FHL2 interacting protein or create a new complex with additional partner(s) as it is often the case in the host-pathogens protein-protein interactions^54^. Interestingly, ElrA is not the only surface-exposed protein that is interacting with host cytoplasmic partners. Indeed, Listeria express at its surface 2 virulence factors implicated in its cytoplasmic survival: ActA (actin assembly-inducing protein) and InlK (Internalin-like protein K) that interact with Arp2/3 and the Major Vault Protein (MVP), respectively^55^. The parallel between ElrA and InlK is interesting, as both proteins are surface-exposed virulence factors and only expressed *in vivo* ^55^. Like FHL2, MVP is implicated in multiple cellular processes but its precise role remains unknown. MVP is recruited at *Listeria* surface impairing autophagic recognition and consequently leading to *Listeria* survival in the cytoplasm^56^. It is tempting to speculate a similar role for ElrA/FHL2 interaction as *E. faecalis* has been recently described to resist autophagy^52^. However the role of ElrA in *E. faecalis* autophagy resistance remains to be investigated.

The FHL2 Interacting Domain (FID) of ElrA is 100% conserved in all the sequenced strains of *E. faecalis* suggesting a crucial functional role of this domain. Among the 24,788 prokaryotic registered genome^57^, apart the *Enterococcus* genus, the FID was detected in an internalin-like protein of *Weissella halotolerans* exhibiting 35% of identity with the FID of ElrA. This *W. halotolerans* protein could be an ElrA-like protein as it shares 34% of identity with ElrA and contains in addition to the FID, an LRR repeat domain and a WxL domain. It is noteworthy that *W. halotolerans* is an opportunistic pathogen for the rainbow trout^58^.

In contrast to the ElrA-deficient strain, we did not find any difference between the wild-type and *elrA*ΔFID strain in the peritoneal infection model (data not shown) suggesting that the FID is not involved in the ElrA-dependent virulence via this infectious entry and that probably other domains of the ElrA protein are important for virulence; but it also demonstrated that the FID-deleted protein is stable and functional. On the opposite, in the UTI model, the impairment of the interaction of ElrA with FHL2 led to a systemic infection but with intermediate *E. faecalis* burdens in mice organs compared to wild-type strain (higher counts) and the fully deleted *elrA* strain (lower counts), revealing that ElrA contributes to virulence in ascending urinary tract infection. Differences in levels of attenuation of *elrA*ΔFID in the models of UTI and SI suggest that the interaction between ElrA and FHL2 is tissue-specific and that other domains of ElrA may be at least as important as the FID to ensure a complete virulence. This observation may be related to the expression pattern of FHL2. It is expressed at basal level in most of the human and mice tissues (in human:<20 fragments per kilobase of exon per million fragments mapped (FPKM)) but shows a higher level of expression in given tissues such as the heart muscle (516 FPKM), the ovary (456 FPKM), the placenta (74 FPKM) and the urinary bladder (90 FPKM)^59^).

Altogether, our study evidenced a strong interaction between ElrA (from *E. faecalis*) and FHL2 (from human) with consequences in 2 infectious models giving new hints to decipher the mode of action of this bacterial effector. This work underlined that the involvement of ElrA in *Enterococcus* virulence could depend on the route of infection. Understanding the functional role and the significance of the interaction of ElrA of *E. faecalis* with FHL2 could provide new insights into the mechanisms of virulence of this poorly understood human pathogen.

## Materials and Methods

### Bacterial and yeast strains, growth conditions, plasmids and oligonucleotides

The strains used in this work are summarized in Table 2. *Escherichia coli* strains were grown at 37 °C in Luria-Bertani (LB) broth or agar medium supplemented with the required antibiotics: ampicillin (100 µg.mL^−1^), spectinomycin (100 µg.mL^−1^), erythromycin (150 µg.mL^−1^). *E. faecalis* strains were grown at 37 °C in M17 broth or agar supplemented with 0.5% of glucose (M17G) and the required antibiotics: tetracycline (5 µg.mL^−1^), erythromycin (30 µg.mL^−1^). S*accharomyces cerevisiae* strains were grown in rich YEPD medium (yeast extract 10 g.L^−1^, peptone 20 g.L^−1^ and dextrose 20 g.L^−1^) or in synthetic complete medium (SC) lacking the amino acids (Leu, Trp and His) or nucleobase (Ade) corresponding to its auxotrophies. The vectors used for the constructs generated in this work are listed in Table 2. The plasmids and primers generated by the cloning of the 23 genes of *E. faecalis* strain V583 and the 39 deletants of the *elrA* gene in the pDONR and pDestDB-GW vectors are listed in Table S1.

### Gateway cloning of full-length *E. faecalis* ORFs and partial *elrA* fragments

The full-length *E. faecalis* selected ORFs (EfORFs) were cloned using the Gateway system (*Invitrogen*) as described in Dobrijevic *et al*.^34^. For the Y2H experiments, all the *Ef*ORFs were transferred from the pDONR223 into the pDestDB-GW^60^ using the LR clonase II (Invitrogen) to generate the yeast vectors expressing the DB-*Ef*ORF fusion proteins. As we used the Gateway recombination method (Invitrogen), the *att*B1.1 (GGGGACAACTTTGTACAAAAAAGTTGGC) and *att*B2.1 (GGGGACAACTTTGTACAAGAAAGTTGGGTC) sequences were added to the 5′ end of each forward and reverse primers, respectively. An amber stop codon in frame with the *elrA* sequence was incorporated in all reverse primers. The PCR products were then cloned in the pDONR223 as previously described^34^.

### Construction of the 3Flag tag-FHL fusions

The FHL CDS were transferred from pDONR223 to the Gateway destination vector pV1899^61^ using the LR recombination cloning, generating the p3flag-FHL2, p3flag-FHL3, p3flag-FHL1 and p3flag-FHL5 vectors.

### High-throughput yeast two-hybrid

Yeast two-hybrid screening was performed as previously described^33^. Yeast matings were performed with Y8800 and Y8930 strains^62^. The pDestDB vectors carrying the *Ef*ORFs were transformed into the Y8930 *MAT*α yeast and selected on solid synthetic complete (SC) medium lacking leucine (SC-Leu). The autoactivating baits were identified by testing the growth of the yeasts containing the pDESTDB-*Ef*ORFs on solid SC medium lacking leucine and histidine (SC-Leu-His) and containing 2 mM of 3-amino-triazole (3-AT); all growing clones were not selected.

Each DB-*Ef*ORF *MAT*α yeast strain was mated with the 12,212 human AD-ORFs pooled into 130 mini-libraries as described by Rual *et al*.^33^ on YEPD agar. After overnight growth at 30 °C, the diploids expressing the *GAL1::HIS3* yeast two-hybrid reporter were selected on SC-Leu-Trp-His plates containing 2 mM 3-AT^63^. *De novo* autoactivators were eliminated using the counter-selectable marker *CYH2* ^60^. Positive colonies were picked for PCR amplification and identification of the interacting proteins by sequencing of the AD- and DB-ORFs. The Human proteins found to interact with the *E. faecalis* proteins were then individually tested against each *Ef*ORF. For this purpose, we mated *MAT*α Y8930 containing individual DB fused to *Ef* ORF and *MAT*a Y8800 containing the vector expressing the AD fused to the interacting human candidates. The plates were incubated overnight at 30 °C on appropriate SC minimal media to select for the expression of *GAL1::HIS3* or *GAL2::ADE2*. To eliminate the yeast background due to residual growth on the minimal media, the plates were twice velvet-cleaned as described^63^. The same procedure was used to test the interaction of ElrA fragments with FHL2. At each step of the interaction screening and validation procedures the following yeast control strains were spotted on the plates: diploids yeasts containing [1] pPC97-CYH (DB-empty) and pPC86 (AD-empty), [2] DB-RB1 and AD-E2F1, [3] DB-fos and AD-jun, [4] pPC97 (DB-empty) and pPC86 (AD-empty), [5] DB-DP1 and AD-E2F1, [6] DB-FRM-3 and AD-PKc_like.

### Cell culture

HEK293T cells (ATCC CRL-3216) and Hela (ATCC CCL-2) were maintained in RPMI 1640 supplemented with 10% heat-inactivated foetal calf serum, 2 mM glutamine, 1X Non essential Amino acid, penicillin (50 U/ml) and streptomycin (50 U/ml) in a humidified 5% CO2 atmosphere at 37 °C. All culture media and supplements were supplied by Life technologies.

### GST pull-down

GST-ErlA fusion proteins (ElrA(27–473), ElrA(27–723) or ElrA(474–606)) were produced in *E. coli* ER266 and purified as previously described^21^. HEK293T cells were transfected with the p3flag-FHL1, p3flag-FHL2, p3flag-FHL3 and p3flag-FHL5 vectors using the jetPrime reagent (Polyplus) following the manufacturer’s instructions. The transfected cells were lysed 10 min on ice in PBS-1% Triton X100-Complete anti-protease cocktail (lysis buffer). Cell debris and nucleus were removed by centrifugation (at 12,000 *g*, 15 min). Then, the cell extracts (200–400 μg of proteins) were incubated overnight at 4 °C with the various GST-ElrA proteins bound to the beads in the lysis buffer. After incubation, the beads were washed six times in the lysis buffer supplemented with 0.5 M NaCl. The original lysates (inputs) and pulled-down proteins were analyzed by Western blotting using the monoclonal mouse anti-FLAG M2 (Sigma-Aldrich 1:2000) or the monoclonal mouse anti-GST (Sigma, 1:2000) as primary antibodies and the HRP-conjugated mouse anti-human IgG as secondary antibody. The Amersham ECL^TM^ Prime Western blotting detection (GE Healthcare) was used for the revelation step.

### Immunofluorescence and confocal microscopy

The FHL2 CDS was transferred by LR from the pDONR223 to the pCMVHA-GW vector^21^ generating the pCMVHA-FHL2 plasmid. Similarly, the myc-ElrA(27–723) and myc-ElrA(27–473) were produced from the pCMVmyc-ElrA vectors generated by LR with pCMVmyc-GW destination vector^64^. The transfection in HeLa cells were performed as described^21^. HeLa cells were grown on coverslips and fixed in 3% paraformaldehyde in PBS, for 15 min. Fixed cells were washed in PBS and permeabilized with 0.5% Triton for 5 min. Coverslips were incubated with primary antibodies in PBS 5% horse serum (i.e., mouse anti-c-myc IgG1κ (clone 9E10, Roche), or rat anti-HA IgG1 (3F10, Roche)) for 30 min at room temperature, washed in PBS 5% horse serum and then incubated with the secondary antibodies (i.e., donkey anti-mouse IgG conjugated to Alexa Fluor 488 (Invitrogen)), and goat anti-rat IgG conjugated to Alexa Fluor 594 (Invitrogen). Coverslips were mounted onto glass slides using Mowiol (Polysciences). Images were acquired using a Zeiss LSM510 laser scanning confocal microscope and analysed using the Zeiss Zen software (Mima2 facilities, Jouy-en-Josas France).

### Construction of the *E. faecalis elrA*ΔFID mutants

Molecular cloning techniques and gel electrophoresis were performed as described previously^65^. To delete FID domain of the *elrA* gene, upstream and downstream regions flanking the domain were amplified by PCR using *E. faecalis* OG1RF genomic DNA as template, and the following primer pairs: (i) *FID*up1 (5′-TTAGTCCGTTAGGTCAAGC-3′) and *FID*up2 (5′-TTCTTTGACAGGTTGGACG-3′); (ii) *FID*down1 (5′-CGTCCAACCTGTCAAAGAAAGTAGCACGACCCAATATC-3′) and *FID*down2 (5′-AATAGCCACGCCTAAAGTG-3′). The two fragments (803 and 815 pb) were purified and a second PCR using *FID*up1 and *FID*down2 allowed the association of the two fragments in a single PCR product that was cloned in pGEM-T Easy (Promega Corporation) to generate pGEM-T-*elrA*ΔFID. After *Eco*RI restriction of the pGEM-T-*elrA*ΔFID, the ΔFID*elrA* fragment was cloned into *Eco*RI-restricted pG^+^host9^66^, generating pG^+^host9-*elrA*ΔFID. The resulting plasmid was then introduced into OG1RF. An in-frame *elrA*ΔFID mutant was selected as previously described^10^ and the deletion of the FID domain was confirmed by sequencing of the chromosomal locus. The procedure was repeated a second time with another pG^+^host9-*elrA*ΔFID vector to obtain an independent *elrA*ΔFID mutant. The mutants were named VE18355–3 and VE18355–4 (Table 2).

### *In vivo* infections

The mouse urinary tract infections (UTIs) experiments were performed by following the protocol described by Lebreton *et al*.^67^. Briefly, groups of 15 female BALB/c mice (10 weeks old; Harlan Italy Srl, San Pietro al Natisone, Udine, Italy) were transurethrally challenged with 1 × 10^4^ colony-forming units (CFUs) of wild-type OG1RF and its derivatives mutants Δ*elrA* and two independent *elrA*ΔFID mutants (Table 2). 48 hours after infection, bladder and kidney pair cultures were prepared from mice that had been humanely killed to determine the recovered CFUs from organ homogenates.

We used a previously well-established systemic intravenous infection (SI) model^68^, the strains were cultured as in the UTI model. A 100 μL aliquot from each strain suspension (1 × 10^9^ bacteria/mL) was injected into the tail vein of each of five female BALB/c mice. Seven days after infection, mice were sacrificed, and their organs (kidneys and livers) were removed aseptically, weighed, and homogenized, to determine the recovered CFUs. The experiments for each animal model were made in triplicate, and comparison among the organ counts was performed by use of an unpaired *t* test.

Mice were observed twice daily for morbidity and mortality, and any mice that exhibited noticeable morbidity (i.e., lethargy, scruffy coats) were euthanized immediately by CO_2_ inhalation. In addition, to minimize animal suffering and distress during the intraurethral catheterization, mice were anesthetized by isoflurane inhalation.

### Statement

All methods were performed in accordance with the relevant guidelines and regulations.

The mouse experiments were approved by the Institutional Animal Use and Care Committee at the Università Cattolica del Sacro Cuore, Rome, Italy (permit number BB21, 04/07/2012), and authorized by the Italian Ministry of Health, according to the Legislative Decree 116/92, which implemented the European Directive 86/609/EEC on laboratory animal protection in Italy. Animal welfare was routinely checked by veterinarians of the Service for Animal Welfare.

## Electronic supplementary material

Supplementary Information
