# Supplementary material for: The Enterococcus faecalis virulence factor ElrA interacts with the human Four-and-a-Half LIM Domains Protein 2
Source: Sci Rep. 2017 Jul 4;7:4581. doi: 10.1038/s41598-017-04875-3 (PMC5496941; doi:10.1038/s41598-017-04875-3)

# Supporting Information

**The *Enterococcus faecalis* virulence factor ElrA interacts with the human Four-and-a-Half LIM Domains Protein 2.**

Alexandre Jamet^1 *^, Rozenn Dervyn^1^, Nicolas Lapaque^1^, Francesca Bugli^2^, Naima G. Perez-Cortez^1 #a^, Hervé M. Blottière^1^, Jean-Claude Twizere^3^, Maurizio Sanguinetti^2^, Brunella Posteraro^4^, Pascale Serror^1^, Emmanuelle Maguin^1^

^1^Micalis Institute, INRA, AgroParisTech, Université Paris-Saclay, 78350 Jouy-en-Josas, France.

^2^Institute of Microbiology, University Cattolica del Sacro Cuore, Rome, Italy.

^3^GIGA Proteins signalisation and interaction, University of Liege, Liege, Belgium.

^4^Institute of Public Health, Section of Hygiene, Universita Cattolica del Sacro Cuore, Rome, Italy.

^#a^Current Address: INRA, Unité d’Immuno-Allergie Alimentaire, iBiTecS/SPI, Gif-sur-Yvette, France.

^*^Correspondence to [alexandre.jamet@inra.fr](mailto:alexandre.jamet@inra.fr)

**S1 Table. Supplementary plasmids and primers used in this study**

The nomenclature used for the various constructs is i) for the 23 *E. faecalis* genes, the name of the plasmid followed by the name of the protein (*eg* pDONR-EF0377) and ii) for the 39 *elrA* deletants, the name of the plasmid followed by the name of the protein and in index the extremities of the protein encoded by the cloned fragment (*e.g.* pDONR-ElrA_1-473_).

| **Plasmid** | **Description** | **Reference or source** |
| --- | --- | --- |
| **pDONR-EF0149** | pDONR223 carrying EF0149 | this study |
| **pDONR-EF0485** | pDONR223 carrying EF0485 | this study |
| **pDONR-EFA0047** | pDONR223 carrying EFA0047 | this study |
| **pDONR-EFB0011** | pDONR223 carrying EFB0011 | this study |
| **pDONR-EF0089** | pDONR223 carrying EF0089 | this study |
| **pDONR-EF0286** | pDONR223 carrying EF0286 | this study |
| **pDONR-EF0377** | pDONR223 carrying EF0377 | this study |
| **pDONR-EF0592** | pDONR223 carrying EF0592 | this study |
| **pDONR-EF0775** | pDONR223 carrying EF0775 | this study |
| **pDONR-EF1091** | pDONR223 carrying EF1091 | this study |
| **pDONR-EF1092** | pDONR223 carrying EF1092 | this study |
| **pDONR-EF1093** | pDONR223 carrying EF1093 | this study |
| **pDONR-EF1099** | pDONR223 carrying EF1099 | this study |
| **pDONR-EF1249** | pDONR223 carrying EF1249 | this study |
| **pDONR-EF1896** | pDONR223 carrying EF1896 | this study |
| **pDONR-EF2090** | pDONR223 carrying EF2090 | this study |
| **pDONR-EF2224** | pDONR223 carrying EF2224 | this study |
| **pDONR-EF2250** | pDONR223 carrying EF2250 | this study |
| **pDONR-EF2505** | pDONR223 carrying EF2505 | this study |
| **pDONR-EF2525** | pDONR223 carrying EF2525 | this study |
| **pDONR-EF2686** | pDONR223 carrying EF2686 (*elrA*) | this study |
| **pDONR-EF2713** | pDONR223 carrying EF2713 | this study |
| **pDONR-EF3314** | pDONR223 carrying EF3314 | this study |
| **pDestDB-EF0149** | pDestDB carrying EF0149 | this study |
| **pDestDB-EF0485** | pDestDB carrying EF0485 | this study |
| **pDestDB-EFA0047** | pDestDB carrying EFA0047 | this study |
| **pDestDB-EFB0011** | pDestDB carrying EFB0011 | this study |
| **pDestDB-EF0089** | pDestDB carrying EF0089 | this study |
| **pDestDB-EF0286** | pDestDB carrying EF0286 | this study |
| **pDestDB-EF0377** | pDestDB carrying EF0377 | this study |
| **pDestDB-EF0592** | pDestDB carrying EF0592 | this study |
| **pDestDB-EF0775** | pDestDB carrying EF0775 | this study |
| **pDestDB-EF1091** | pDestDB carrying EF1091 | this study |
| **pDestDB-EF1092** | pDestDB carrying EF1092 | this study |
| **pDestDB-EF1093** | pDestDB carrying EF1093 | this study |
| **pDestDB-EF1099** | pDestDB carrying EF1099 | this study |
| **pDestDB-EF1249** | pDestDB carrying EF1249 | this study |
| **pDestDB-EF1896** | pDestDB carrying EF1896 | this study |
| **pDestDB-EF2090** | pDestDB carrying EF2090 | this study |
| **pDestDB-EF2224** | pDestDB carrying EF2224 | this study |
| **pDestDB-EF2250** | pDestDB carrying EF2250 | this study |
| **pDestDB-EF2505** | pDestDB carrying EF2505 | this study |
| **pDestDB-EF2525** | pDestDB carrying EF2525 | this study |
| **pDestDB-EF2686** | pDestDB carrying EF2686 (*elrA*) | this study |
| **pDestDB-EF2713** | pDestDB carrying EF2713 | this study |
| **pDestDB-EF3314** | pDestDB carrying EF3314 | this study |
| **pDONR-ElrA_1-723_** | pDONR223 carrying *elrA* (amino acid 1-723) amplified with primers ElrA_F1 and ElrA_R1 | this study |
| **pDONR-ElrA_1-473_** | pDONR223 carrying the *elrA*fragment (corresponding to amino acid 1 to 473) amplified with primers ElrA_F1 and ElrA_R2 | this study |
| **pDONR-ElrA_1-387_** | pDONR223 carrying the *elrA*fragment (corresponding to amino acid 1 to 387) amplified with primers ElrA_F1 and ElrA_R3 | this study |
| **pDONR-ElrA_1-141_** | pDONR223 carrying the *elrA*fragment (corresponding to amino acid 1 to 141) amplified with primers ElrA_F1 and ElrA_R4 | this study |
| **pDONR-ElrA_1-60_** | pDONR223 carrying the *elrA*fragment (corresponding to amino acid 1 to 60) amplified with primers ElrA_F1 and ElrA_R5 | this study |
| **pDONR-ElrA_1-26_** | pDONR223 carrying the *elrA*fragment (corresponding to amino acid 1 to 26) amplified with primers ElrA_F1 and ElrA_R6 | this study |
| **pDONR-ElrA_27-723_** | pDONR223 carrying the *elrA*fragment (corresponding to amino acid 27 to 723) amplified with primers ElrA_F2 and ElrA_R1 | this study |
| **pDONR-ElrA_27-473_** | pDONR223 carrying the *elrA*fragment (corresponding to amino acid 27 to 473) amplified with primers ElrA_F2 and ElrA_R2 | this study |
| **pDONR-ElrA_27-387_** | pDONR223 carrying the *elrA*fragment (corresponding to amino acid 27 to 387) amplified with primers ElrA_F2 and ElrA_R3 | this study |
| **pDONR-ElrA2_7-141_** | pDONR223 carrying the *elrA*fragment (corresponding to amino acid 27 to 141) amplified with primers ElrA_F2 and ElrA_R4 | this study |
| **pDONR-ElrA_27-60_** | pDONR223 carrying the *elrA*fragment (corresponding to amino acid 27 to 60) amplified with primers ElrA_F2 and ElrA_R5 | this study |
| **pDONR-ElrA_61-723_** | pDONR223 carrying the *elrA*fragment (corresponding to amino acid 61 to 723) amplified with primers ElrA_F3 and ElrA_R1 | this study |
| **pDONR-ElrA_61-473_** | pDONR223 carrying the *elrA*fragment (corresponding to amino acid 61 to 473) amplified with primers ElrA_F3 and ElrA_R2 | this study |
| **pDONR-ElrA_61-387_** | pDONR223 carrying the *elrA*fragment (corresponding to amino acid 61 to 387) amplified with primers ElrA_F3 and ElrA_R3 | this study |
| **pDONR-ElrA_61-141_** | pDONR223 carrying the *elrA*fragment (corresponding to amino acid 61 to 141) amplified with primers ElrA_F3 and ElrA_R4 | this study |
| **pDONR-ElrA_142-723_** | pDONR223 carrying the *elrA*fragment (corresponding to amino acid 142 to 723) amplified with primers ElrA_F4 and ElrA_R1 | this study |
| **pDONR-ElrA_142-473_** | pDONR223 carrying the *elrA*fragment (corresponding to amino acid 142 to 473) amplified with primers ElrA_F4 and ElrA_R2 | this study |
| **pDONR-ElrA_142-387_** | pDONR223 carrying the *elrA*fragment (corresponding to amino acid 142 to 387) amplified with primers ElrA_F4 and ElrA_R3 | this study |
| **pDONR-ElrA_388-723_** | pDONR223 carrying the *elrA*fragment (corresponding to amino acid 388 to 723) amplified with primers ElrA_F5 and ElrA_R1 | this study |
| **pDONR-ElrA_388-473_** | pDONR223 carrying the *elrA*fragment (corresponding to amino acid 388 to 473) amplified with primers ElrA_F5 and ElrA_R2 | this study |
| **pDONR-ElrA_388-606_** | pDONR223 carrying the *elrA*fragment (corresponding to amino acid 388 to 606) amplified with primers ElrA_F5 and ElrA_R1.6 | this study |
| **pDONR-ElrA_388-625_** | pDONR223 carrying the *elrA*fragment (corresponding to amino acid 388 to 625) amplified with primers ElrA_F5 and ElrA_R1.5 | this study |
| **pDONR-ElrA_474-723_** | pDONR223 carrying the *elrA*fragment (corresponding to amino acid 474 to 723) amplified with primers ElrA_F6 and ElrA_R1 | this study |
| **pDONR-ElrA_474-701_** | pDONR223 carrying the *elrA* fragment (corresponding to amino acid 474 to 701) amplified with primers ElrA_F6 and ElrA_R1.2 | this study |
| **pDONR-ElrA_474-682_** | pDONR223 carrying the *elrA* fragment (corresponding to amino acid 474 to 682) amplified with primers ElrA_F6 and ElrA_R1.3 | this study |
| **pDONR-ElrA_474-644_** | pDONR223 carrying the *elrA* fragment (corresponding to amino acid 474 to 644) amplified with primers ElrA_F6 and ElrA_R1.4 | this study |
| **pDONR-ElrA_474-625_** | pDONR223 carrying the *elrA* fragment (corresponding to amino acid 474 to 625) amplified with primers ElrA_F6 and ElrA_R1.5 | this study |
| **pDONR-ElrA_474-606_** | pDONR223 carrying the *elrA* fragment (corresponding to amino acid 474 to 606) amplified with primers ElrA_F6 and ElrA_R1.6 | this study |
| **pDONR-ElrA_474-568_** | pDONR223 carrying the *elrA* fragment (corresponding to amino acid 474 to 568) amplified with primers ElrA_F6 and ElrA_R1.7 | this study |
| **pDONR-ElrA_474-530_** | pDONR223 carrying the *elrA* fragment (corresponding to amino acid 474 to 530) amplified with primers ElrA_F6 and ElrA_R1.8 | this study |
| **pDONR-ElrA_474-592_** | pDONR223 carrying the *elrA* fragment (corresponding to amino acid 474 to 492) amplified with primers ElrA_F6 and ElrA_R1.9 | this study |
| **pDONR-ElrA_512-723_** | pDONR223 carrying the *elrA* fragment (corresponding to amino acid 512 to 723) amplified with primers ElrA_F6.2 and ElrA_R1 | this study |
| **pDONR-ElrA_550-723_** | pDONR223 carrying the *elrA* fragment (corresponding to amino acid 550 to 723) amplified with primers ElrA_F6.3 and ElrA_R1 | this study |
| **pDONR-ElrA_588-723_** | pDONR223 carrying the *elrA* fragment (corresponding to amino acid 588 to 723) amplified with primers ElrA_F6.4 and ElrA_R1 | this study |
| **pDONR-ElrA_607-723_** | pDONR223 carrying the *elrA* fragment (corresponding to amino acid 607 to 723) amplified with primers ElrA_F6.5 and ElrA_R1 | this study |
| **pDONR-ElrA_626-723_** | pDONR223 carrying the *elrA* fragment (corresponding to amino acid 626 to 723) amplified with primers ElrA_F6.6 and ElrA_R1 | this study |
| **pDONR-ElrA_645-723_** | pDONR223 carrying the *elrA* fragment (corresponding to amino acid 645 to 723) amplified with primers ElrA_F6.7 and ElrA_R1 | this study |
| **pDONR-ElrA_683-723_** | pDONR223 carrying the *elrA* fragment (corresponding to amino acid 683 to 723) amplified with primers ElrA_F6.8 and ElrA_R1 | this study |
| **pDONR-ElrA_702-723_** | pDONR223 carrying the *elrA* fragment (corresponding to amino acid 702 to 723) amplified with primers ElrA_F6.9 and ElrA_R1 | this study |
| **pDB-ElrA_1-723_** | pDestDB carrying *elrA* transfered by LR reaction from pDON-ElrA_1-723_ | this study |
| **pDB-ElrA_1-473_** | pDestDB carrying *elrA* transfered by LR reaction from pDON-ElrA_1-473_ | this study |
| **pDB-ElrA_1-387_** | pDestDB carrying *elrA* transfered by LR reaction from pDON-ElrA_1-387_ | this study |
| **pDB-ElrA_1-141_** | pDestDB carrying *elrA* transfered by LR reaction from pDON-ElrA_1-141_ | this study |
| **pDB-ElrA_1-60_** | pDestDB carrying *elrA* transfered by LR reaction from pDON-ElrA_1-60_ | this study |
| **pDB-ElrA_1-26_** | pDestDB carrying *elrA* transfered by LR reaction from pDON-ElrA_1-26_ | this study |
| **pDB-ElrA_27-723_** | pDestDB carrying *elrA* transfered by LR reaction from pDON-ElrA_27-723_ | this study |
| **pDB-ElrA_27-473_** | pDestDB carrying *elrA* transfered by LR reaction from pDON-ElrA_27-473_ | this study |
| **pDB-ElrA_27-387_** | pDestDB carrying *elrA* transfered by LR reaction from pDON-ElrA_27-387_ | this study |
| **pDB-ElrA_27-141_** | pDestDB carrying *elrA* transfered by LR reaction from pDON-ElrA_27-141_ | this study |
| **pDB-ElrA_27-60_** | pDestDB carrying *elrA* transfered by LR reaction from pDON-ElrA_27-60_ | this study |
| **pDB-ElrA_61-723_** | pDestDB carrying *elrA* transfered by LR reaction from pDON-ElrA_61-723_ | this study |
| **pDB-ElrA_61-473_** | pDestDB carrying *elrA* transfered by LR reaction from pDON-ElrA_61-473_ | this study |
| **pDB-ElrA_61-387_** | pDestDB carrying *elrA* transfered by LR reaction from pDON-ElrA_61-387_ | this study |
| **pDB-ElrA_61-141_** | pDestDB carrying *elrA* transfered by LR reaction from pDON-ElrA_61-141_ | this study |
| **pDB-ElrA_142-723_** | pDestDB carrying *elrA* transfered by LR reaction from pDON-ElrA_142-723_ | this study |
| **pDB-ElrA_142-473_** | pDestDB carrying *elrA* transfered by LR reaction from pDON-ElrA_142-473_ | this study |
| **pDB-ElrA_142-387_** | pDestDB carrying *elrA* transfered by LR reaction from pDON-ElrA_142-387_ | this study |
| **pDB-ElrA_388-723_** | pDestDB carrying *elrA* transfered by LR reaction from pDON-ElrA_388-723_ | this study |
| **pDB-ElrA_388-473_** | pDestDB carrying *elrA* transfered by LR reaction from pDON-ElrA_388-473_ | this study |
| **pDB-ElrA_474-723_** | pDestDB carrying *elrA* transfered by LR reaction from pDON-ElrA_474-723_ | this study |
| **pDB-ElrA_474-701_** | pDestDB carrying *elrA* transfered by LR reaction from pDON-ElrA_474-701_ | this study |
| **pDB-ElrA_474-682_** | pDestDB carrying *elrA* transfered by LR reaction from pDON-ElrA_474-682_ | this study |
| **pDB-ElrA_474-644_** | pDestDB carrying *elrA* transfered by LR reaction from pDON-ElrA_474-644_ | this study |
| **pDB-ElrA_474-625_** | pDestDB carrying *elrA* transfered by LR reaction from pDON-ElrA_474-625_ | this study |
| **pDB-ElrA_474-606_** | pDestDB carrying *elrA* transfered by LR reaction from pDON-ElrA_474-606_ | this study |
| **pDB-ElrA_474-568_** | pDestDB carrying *elrA* transfered by LR reaction from pDON-ElrA_474-568_ | this study |
| **pDB-ElrA_474-530_** | pDestDB carrying *elrA* transfered by LR reaction from pDON-ElrA_474-530_ | this study |
| **pDB-ElrA_474-492_** | pDestDB carrying *elrA* transfered by LR reaction from pDON-ElrA_474-492_ | this study |
| **pDB-ElrA_512-723_** | pDestDB carrying *elrA* transfered by LR reaction from pDON-ElrA_512-723_ | this study |
| **pDB-ElrA_550-723_** | pDestDB carrying *elrA* transfered by LR reaction from pDON-ElrA_550-723_ | this study |
| **pDB-ElrA_588-723_** | pDestDB carrying *elrA* transfered by LR reaction from pDON-ElrA_588-723_ | this study |
| **pDB-ElrA_607-723_** | pDestDB carrying *elrA* transfered by LR reaction from pDON-ElrA_607-723_ | this study |
| **pDB-ElrA_626-723_** | pDestDB carrying *elrA* transfered by LR reaction from pDON-ElrA_626-723_ | this study |
| **pDB-ElrA_645-723_** | pDestDB carrying *elrA* transfered by LR reaction from pDON-ElrA_645-723_ | this study |
| **pDB-ElrA_683-723_** | pDestDB carrying *elrA* transfered by LR reaction from pDON-ElrA_683-723_ | this study |
| **pDB-ElrA_702-723_** | pDestDB carrying *elrA* transfered by LR reaction from pDON-ElrA_702-723_ | this study |
| **Primers used in this study** |  |  |
| **Primer name** | **Sequence 5' to 3'** | **Reference or source** |
| **EF0149_f** | AGGAGGCTCTTCA*ATGAATCAACAGACTGAAGTAA* | this study |
| **EF0149_r** | GAAAGTTGGGTC*CTATTTCACTTCACGTTTTTT* | this study |
| **EF0485_f** | AGGAGGCTCTTCA*ATGAAGCAACAAATGAAAG* | this study |
| **EF0485_r** | GAAAGTTGGGTC*CTATTTTGTTTCTTTTCTACGTT* | this study |
| **EFA0047_f** | AGGAGGCTCTTCA*ATGAAGCAACAAACAGAAG* | this study |
| **EFA0047_r** | GAAAGTTGGGTC*CTATTTTGTTTCTTTTCTACGTT* | this study |
| **EFB0011_f** | AGGAGGCTCTTCA*ATGAATCAACAGACTGAAGTAA* | this study |
| **EFB0011_r** | GAAAGTTGGGTC*CTATTTTGTTTCTTTTCTACGTT* | this study |
| **EF0089_f** | AGGAGGCTCTTCA*ATGAAGCAAACCAGCCAG* | this study |
| **EF0089_r** | GAAAGTTGGGTC*CTATTTCTTGCTCTTTTTATAGAA* | this study |
| **EF0286_f** | AGGAGGCTCTTCA*ATGAACCCAACTATTCAAC* | this study |
| **EF0286_r** | GAAAGTTGGGTC*CTATTTCACAGCCTTAACATT* | this study |
| **EF0377_f** | AGGAGGCTCTTCA*ATGAAGACCTATGAAGTGG* | this study |
| **EF0377_r** | GAAAGTTGGGTC*CTAGTTGTACTGTGCTAAAATTTT* | this study |
| **EF0592_f** | AGGAGGCTCTTCA*ATGTTTTTTACTGGTAAAGAAAG* | this study |
| **EF0592_r** | GAAAGTTGGGTC*CTAATTCGTATTATTTTTTCTTTTT* | this study |
| **EF0775_f** | AGGAGGCTCTTCA*ATGTTTAAGAAAGCAACGA* | this study |
| **EF0775_r** | GAAAGTTGGGTC*CTAATTTTTTCTTTTTTTATTTTTT* | this study |
| **EF1091_f** | AGGAGGCTCTTCA*ATGATAACAGATGAGAATGATAA* | this study |
| **EF1091_r** | GAAAGTTGGGTC*CTACACACTCCCTTCTGG* | this study |
| **EF1092_f** | AGGAGGCTCTTCA*ATGAAAAACGCACGTTGG* | this study |
| **EF1092_r** | GAAAGTTGGGTC*CTATTTTCTCTCTCCTCTTTTT* | this study |
| **EF1093_f** | AGGAGGCTCTTCA*ATGAAGCAATTAAAAAAAGTTT* | this study |
| **EF1093_r** | GAAAGTTGGGTC*CTAAGCATTTTCTTTTCTACG* | this study |
| **EF1099_f** | AGGAGGCTCTTCA*ATGACAAAAAGTGTAAAATTTTT* | this study |
| **EF1099_r** | GAAAGTTGGGTC*CTAATTCTTTCTGATTTGTAGATAAC* | this study |
| **EF1249_f** | AGGAGGCTCTTCA*ATGTCATTTGATGGCGTA* | this study |
| **EF1249_r** | GAAAGTTGGGTC*CTAGGAAGCAGAAGCCTT* | this study |
| **EF1896_f** | AGGAGGCTCTTCA*ATGATAAAGCCGATATTTAAA* | this study |
| **EF1896_r** | GAAAGTTGGGTC*CTAGTTACGGCGATTCCA* | this study |
| **EF2090_f** | AGGAGGCTCTTCA*ATGAGGGTTAGAAGCTTTT* | this study |
| **EF2090_r** | GAAAGTTGGGTC*CTATCTTATCCATGTTGCAC* | this study |
| **EF2224_f** | AGGAGGCTCTTCA*ATGAATAAAGCAGTTAAAAATTT* | this study |
| **EF2224_r** | GAAAGTTGGGTC*CTACTCCTCTTTTTCTTTGTTT* | this study |
| **EF2250_f** | AGGAGGCTCTTCA*ATGGAATTAACAACAACGA* | this study |
| **EF2250_r** | GAAAGTTGGGTC*CTATGGTGTATCATCTAACGAC* | this study |
| **EF2505_f** | AGGAGGCTCTTCA*ATGAAGAAAAAAACTTTTTCTT* | this study |
| **EF2505_r** | GAAAGTTGGGTC*CTATTTTCGTTTCTTCTTGAT* | this study |
| **EF2525_f** | AGGAGGCTCTTCA*ATGAAGAAAACAACGATTATT* | this study |
| **EF2525_r** | GAAAGTTGGGTC*CTAGCCCTCTTTGCTATCT* | this study |
| **EF2686_f** | AGGAGGCTCTTCA*ATGAAAAAAATGTGCATCT* | this study |
| **EF2686_r** | GAAAGTTGGGTC*CTATTTTGCACTGCCATC* | this study |
| **EF2713_f** | AGGAGGCTCTTCA*ATGGAAAAGTCAACAGTTACT* | this study |
| **EF2713_r** | GAAAGTTGGGTC*CTATTTCTTTTTATTTTTTATAATGAAT* | this study |
| **EF3314_f** | AGGAGGCTCTTCA*ATGATCTTGGTATTTATCGTTT* | this study |
| **EF3314_r** | GAAAGTTGGGTC*CTAGTTTTTACGTTTGCGT* | this study |
| **ATTB1.1** | GGGGACAACTTTGTACAAAAAAGTTGGCAAAGGAGGCTCTTCAAT | (34) |
| **ATTB2.1** | GGGGACAACTTTGTACAAGAAAGTTGGGTCCTA | (34) |
| **ElrA_F1** | GGGGACAACTTTGTACAAAAAAGTTGGC*ATGAAAAAAATGTGCATCT* | this study |
| **ElrA_F2** | GGGGACAACTTTGTACAAAAAAGTTGGC*GAAACGACCGAAACAATC* | this study |
| **ElrA_F3** | GGGGACAACTTTGTACAAAAAAGTTGGC*ACCAGTAGTTCGGAGATTC* | this study |
| **ElrA_F4** | GGGGACAACTTTGTACAAAAAAGTTGGC*AAACTGAATAAAGTTATTTTAGAAAA* | this study |
| **ElrA_F5** | GGGGACAACTTTGTACAAAAAAGTTGGC*CAAATGATTATCTTATCTTCTGTC* | this study |
| **ElrA_F6** | GGGGACAACTTTGTACAAAAAAGTTGGC*TTAAGTGAAACGAAACTATCTT* | this study |
| **ElrA_R1** | GGGGACAACTTTGTACAAGAAAGTTGGGTC*CTATTTTGCACTGCCATC* | this study |
| **ElrA_R2** | GGGGACAACTTTGTACAAGAAAGTTGGGTC*CTAGAAAACTTCTTTGACAGGTT* | this study |
| **ElrA_R3** | GGGGACAACTTTGTACAAGAAAGTTGGGTC*CTAATTAGATACATCTAGCCCTGTAA* | this study |
| **ElrA_R4** | GGGGACAACTTTGTACAAGAAAGTTGGGTC*CTAATCCAATTCTGCTTGGGT* | this study |
| **ElrA_R5** | GGGGACAACTTTGTACAAGAAAGTTGGGTC*CTATGCTGAATTTTGCTCTTC* | this study |
| **ElrA_R6** | GGGGACAACTTTGTACAAGAAAGTTGGGTC*CTAGGCGAAAGTTGTTACAGG* | this study |
| **ElrA_F6.2** | GGGGACAACTTTGTACAAAAAAGTTGGC*GATGCTGGGAATAGATTACA* | this study |
| **ElrA_F6.3** | GGGGACAACTTTGTACAAAAAAGTTGGC*CCAGAACTAACTTATGGAAAA* | this study |
| **ElrA_F6.4** | GGGGACAACTTTGTACAAAAAAGTTGGC*GTACCCACAGCGATTTCC* | this study |
| **ElrA_F6.5** | GGGGACAACTTTGTACAAAAAAGTTGGC*TATCCAATTGTTTCCATG* | this study |
| **ElrA_F6.6** | GGGGACAACTTTGTACAAAAAAGTTGGC*AATAATTGGTCGCTTGCA* | this study |
| **ElrA_F6.7** | GGGGACAACTTTGTACAAAAAAGTTGGC*ACGTTGCCGAATATTTTA* | this study |
| **ElrA_F6.8** | GGGGACAACTTTGTACAAAAAAGTTGGC*GATCAATGGAAAGAAAATG* | this study |
| **ElrA_F6.9** | GGGGACAACTTTGTACAAAAAAGTTGGC*AAAGCAGAAGAATACGAAG* | this study |
| **ElrA_R1.2** | GGGGACAACTTTGTACAAGAAAGTTGGGTC*CTAGGCCGTCCCTCCAGGAAC* | this study |
| **ElrA_R1.3** | GGGGACAACTTTGTACAAGAAAGTTGGGTC*CTAAGAAATATTTGTTTCTTGATGTC* | this study |
| **ElrA_R1.4** | GGGGACAACTTTGTACAAGAAAGTTGGGTC*CTAAGCACCTGATTCAGATTT* | this study |
| **ElrA_R1.5** | GGGGACAACTTTGTACAAGAAAGTTGGGTC*CTACCCCGTTTGACGAGTATC* | this study |
| **ElrA_R1.6** | GGGGACAACTTTGTACAAGAAAGTTGGGTC*CTATTGGGTCGTGCTACTAGC* | this study |
| **ElrA_R1.7** | GGGGACAACTTTGTACAAGAAAGTTGGGTC*CTAGTAGCCTTTATCATAGACTTCC* | this study |
| **ElrA_R1.8** | GGGGACAACTTTGTACAAGAAAGTTGGGTC*CTATTTCTGGATTTCTTGACC* | this study |
| **ElrA_R1.9** | GGGGACAACTTTGTACAAGAAAGTTGGGTC*CTATTTTTCCATCGACTTTTC* | this study |
| ***FID*up1** | TTAGTCCGTTAGGTCAAGC | this study |
| ***FID*up2** | TTCTTTGACAGGTTGGACG | this study |
| ***FID*down1** | CGTCCAACCTGTCAAAGAAAGTAGCACGACCCAATATC | this study |
| ***FID*down2** | AATAGCCACGCCTAAAGTG | this study |

**S2 Figure. Full-length blot used in Figure 4.**

The M2 anti-Flag antibody was used to detect the 3Flag-FHL2.


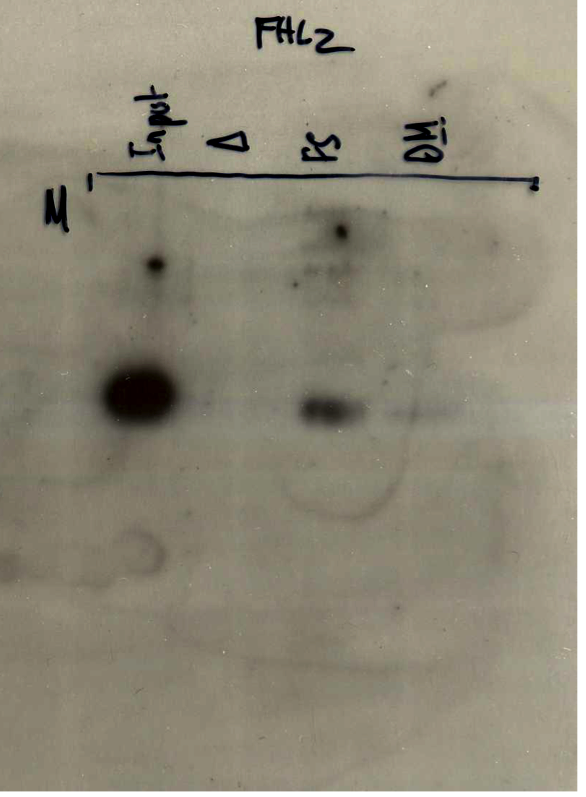

Supplement: Supplementary file 1 — Supplementary Information [file 41598_2017_4875_MOESM1_ESM.doc]
